# Disadvantageous Decision-Making as a Predictor of Drop-Out among Cocaine-Dependent Individuals in Long-Term Residential Treatment

**DOI:** 10.3389/fpsyt.2013.00149

**Published:** 2013-11-15

**Authors:** Laura Stevens, Patricia Betanzos-Espinosa, Cleo L. Crunelle, Esperanza Vergara-Moragues, Herbert Roeyers, Oscar Lozano, Geert Dom, Francisco Gonzalez-Saiz, Wouter Vanderplasschen, Antonio Verdejo-García, Miguel Pérez-García

**Affiliations:** ^1^Department of Orthopedagogics, Ghent University, Ghent, Belgium; ^2^Department of Clinical Psychology, Universidad de Granada, Granada, Spain; ^3^Toxicological Centre, Antwerp University, Antwerp, Belgium; ^4^Collaborative Antwerp Psychiatric Research Institute, Antwerp University, Antwerp, Belgium; ^5^Department of Education, International University of La Rioja (UNIR), Madrid, Spain; ^6^Red de Trastornos Adictivos, Universidad de Granada, Granada, Spain; ^7^Department of Experimental Clinical and Health Psychology, Ghent University, Ghent, Belgium; ^8^Department of Psychology, Universidad de Huelva, Huelva, Spain; ^9^Psychiatric Centre Alexian Brothers, Boechout, Belgium; ^10^Unidad de Salud Mental Comunitaria Villamartín, Unidad de Gestión Clínica Hospital de Jerez, Cádiz, Spain; ^11^School of Psychology and Psychiatry, Monash University, Melbourne, VIC, Australia; ^12^Institute of Neuroscience F. Olóriz, Universidad de Granada, Granada, Spain; ^13^Mind, Brain and Behavior Research Center, Universidad de Granada, Granada, Spain

**Keywords:** decision-making, drop-out, treatment retention, addiction treatment outcomes, cocaine dependence

## Abstract

**Background**: The treatment of cocaine-dependent individuals (CDI) is substantially challenged by high drop-out rates, raising questions regarding contributing factors. Recently, a number of studies have highlighted the potential of greater focus on the clinical significance of neurocognitive impairments in treatment-seeking cocaine users. In the present study, we hypothesized that disadvantageous decision-making would be one such factor placing CDI at greater risk for treatment drop-out.

**Methods**: In order to explore this hypothesis, the present study contrasted baseline performance (at treatment onset) on two validated tasks of decision-making, the Iowa Gambling Task (IGT) and the Cambridge Gamble Task (CGT) in CDI who completed treatment in a residential Therapeutic Community (TC) (*N* = 66) and those who dropped out of TC prematurely (*N* = 84).

**Results**: Compared to treatment completers, CDI who dropped out of TC prematurely did not establish a consistent and advantageous response pattern as the IGT progressed and exhibited a poorer ability to choose the most likely outcome on the CGT. There were no group differences in betting behavior.

**Conclusion**: Our findings suggest that neurocognitive rehabilitation of disadvantageous decision-making may have clinical benefits in CDI admitted to long-term residential treatment programs.

## Introduction

The treatment of cocaine-dependent individuals (CDI) is substantially challenged by high drop-out rates. Whereas treatment attrition is high across the majority of substance abuse treatment studies, drop-out rates ranging from 60 to 80% have been reported among CDI (1–3). These high drop-out rates are particularly problematic, given the well-established association between the length of time spent in treatment (i.e., treatment retention) and post-treatment outcomes. More specifically, a sufficient length of time spent in the treatment program constitutes one of the strongest and most consistent predictors of positive post-treatment outcomes, including sustained abstinence (4, 5). Conversely, CDI who drop-out of treatment prematurely fare worse than those who stay in treatment for the entire period: high drop-out rates limit overall treatment effectiveness, increase the propensity to relapse and seriously exacerbate health, financial, and legal consequences (6, 7). This relationship between treatment drop-out/completion and post-treatment outcomes has been found across all the major addiction treatment modalities (8–10), including drug-free inpatient therapeutic communities (TCs), which remain a core modality of the drug treatment system in Europe and the United States (11, 12).

The high attrition rates observed among CDI and their detrimental consequences raise questions regarding contributing factors that might influence treatment drop-out in this population. Finding a way to predict premature treatment drop-out could help in the early identification of CDI with the highest risk for drop-out, such that these individuals may receive additional monitoring and adequate therapeutic interventions targeting specific risk factors.

A recent generation of research, facilitated by considerable advances in the field of neuroscience, has begun to examine whether neurocognitive impairments in CDI may confer an increased risk of drop-out (13, 14). Indeed, growing evidence indicating that a substantial number of CDI suffer from detectable damage in cortical and sub-cortical brain regions and exhibit deficits across a range of neurocognitive domains (15, 16) has recently encouraged researchers to focus on neurocognitive factors when attempting to predict treatment drop-out. Although preliminary, these studies seem to suggest that CDI who drop-out of treatment prematurely demonstrate significantly poorer performance than treatment completers across various cognitive domains, including attention, memory, and processing speed (13, 14, 17). As such, intact executive functioning may be a necessary prerequisite to successfully complete treatment or attain treatment objectives.

Surprisingly, very few studies have focused on the prognostic utility of more specific aspects of neurocognitive functioning, such as those related to the domain of (affective) decision-making (18, 19). This lack of research is particularly striking given the well-established role of impaired decision-making in the pathogenesis and pathophysiology of addiction (20, 21). A substantial number of drug-dependent individuals shows behavioral signs of disadvantageous decision-making, characterized by a preference for immediate rewards while disregarding long-term consequences (a pattern coined “myopia for the future”), despite these choices being less adaptive with regard to overall expected value (22, 23). For example, neurocognitive assessment using the Iowa Gambling Task (IGT) (24) has shown that drug-dependent individuals are more likely to make maladaptive decisions, resulting in long-term losses exceeding short-term gains (25). Similarly, evidence suggests that a number of drug-dependent individuals fail to improve their performance on this task based on trial-by-trial outcomes (26). Using alternative probes of decision-making which minimize learning requirements (i.e., decision-making under risk rather than under ambiguous conditions), other studies showed that drug-dependent individuals demonstrated an increased tendency to choose the less likely outcome, despite having processed information regarding outcome probabilities (27).

Over the years, numerous studies have established the ecological and predictive validity of disadvantageous decision-making in drug users (26, 28, 29). In particular, poor decision-making in drug-dependent individuals has shown significant correlations with real-life everyday functioning, including social impairment, problems with maintaining gainful employment, and difficulties with achieving and maintaining substantial periods of abstinence (26, 28, 30–32). Hypothetically, a decision-making style characterized by impaired integration of affective/cognitive information into future strategies (i.e., poor learning from experience) or an immediate reward preference disregarding long-term future consequences may also put CDI at greater risk for premature treatment drop-out. However, despite the intuitive appeal of such a relationship, the association between poor decision-making and premature treatment drop-out among CDI has remained underexplored.

To the best of our knowledge, only two studies – including one of our own research group – have examined the relationship between disadvantageous decision-making and treatment outcomes in CDI (19, 32). Both studies used the length of stay in treatment as the outcome variable of interest and found that disadvantageous decision-making, as indexed by lower IGT net scores, was unrelated to treatment retention among these individuals (19, 32). However, treatment retention and drop-out have recently been found to be predicted by different variables (11) and as such, it remains unknown whether and how disadvantageous decision-making in CDI relates to treatment drop-out. Further, by selectively focusing on overall IGT net scores, previous retention studies did not differentiate between distinct components of decision-making.

With the present study, we aimed to refine our initial findings by introducing a number of relevant novelties compared to previous research: first, we used treatment drop-out (rather than the number of days in treatment) as the outcome variable of interest. Further, to better parse some important components of decision-making that may be relevant to treatment drop-out, the present study utilized two complementary decision-making measures: the IGT, which factors reward/punishment-based decision-making learning, and the Cambridge Gamble Task (CGT) (27), which factors risk-based decision-making outside a learning context. We hypothesized that impaired decision-making, as indexed by (1) a failure to develop a preference for the advantageous decks during the course of the IGT and (2) poor decision-making on the CGT, would be associated with treatment drop-out among primarily CDI admitted to residential TCs.

## Materials and Methods

### Participants

Eligible participants were recruited from six different TCs located in the region of Andalusia (Spain): Cartaya, Almonte, Mijas, Los Palacios, La Línea, and Tarifa. All TCs had a common treatment program that is based on multidisciplinary interventions, including Cognitive Behavioral Therapy (CBT), psycho-education, and occupational therapy. More details regarding the recruitment context of this study have been described elsewhere [see Verdejo-Garcia et al. (29)]. For inclusion, participants had to (1) meet the DSM-IV-TR for cocaine dependence and report cocaine as their primary substance of abuse, (2) be able to understand test instructions and perform the neuropsychological assessment, and (3) be abstinent for at least 15 days (in order to avoid potential effects of acute intoxication or withdrawal symptoms on neurocognitive task performance). Individuals meeting the criteria for nicotine or heroin dependence and/or alcohol abuse were also included. Exclusion criteria included dependence on other substances (e.g., other opioids, benzodiazepines, cannabinoids, barbiturates, hallucinogenics) and being abstinent for more than 2 months. DSM-criteria were determined using the Spanish version of the Psychiatric Research Interview for Substance and Mental Disorders (33). Information about the frequency, amount, and duration of drug use was collected using the Interview for Research on Addictive Behavior (IRAB) (34).

### Assessment procedure

After the clinical staff had screened potential participants for inclusion criteria, individuals were informed about the aims of the study and provided written informant consent. The study was approved by the Comité de Ética en Investigación Humana (CEIH) of the University of Granada. A baseline neuropsychological assessment was performed between day 20 and 30 following treatment entry. Assessment of decision-making was undertaken by an experienced neuropsychologist in a quiet testing environment in each of the six different TCs.

### Decision-making assessment

The IGT is a computer task that requires individuals to choose from four decks of cards, decks A, B, C, and D. Unbeknownst to the participants, two decks (i.e., A and B) are associated with large wins but even larger losses (resulting in net loss), whereas the other two decks (i.e., C and D) are associated with smaller wins but also smaller losses (resulting in overall profit). During the course of the task, healthy participants usually develop a preference for the safe decks (C and D). In contrast, individuals with impaired decision-making often continue to choose cards from the risky decks (A and B), which in the long run, will take more money than they give. The 100 trials were grouped into five blocks of 20 consecutive cards, with a net score for each block calculated as (C + D) − (A + B) decks. Calculating net scores for each block of 20 trials permits an analysis of learning across the different phases of the IGT. An overall IGT net score was also determined by adding up the individual block scores. Selecting more cards from bad decks results in an overall net loss across the 100 trials of the task, whereas choosing more cards from the good decks results in overall net gains.

The Cambridge Gamble Task (CGT) of the *CANTABeclipse* Battery is a computerized task in which participants are presented with a row of 10 boxes at the top of the screen, each of which can be either red or blue. At the bottom of the screen are rectangles containing the words “Red” and “Blue.” Participants are instructed to guess whether a yellow token is hidden in a red box or a blue box. After making a choice, participants are asked to place a bet on this choice being correct. Available bets are offered in a sequence, as a proportion of the participant’s points total on that trial (ascending condition: 5, 25, 50, 75, 95%; descending condition: 95, 75, 50, 25, 5%). After the bet is placed, the hidden token is revealed and the bet is added to or subtracted from the total score. Dependent measures were (1) quality of decision-making (i.e., the percentage of trials subjects bet on the more likely outcome), (2) risk-taking (the mean proportion of current points total that the subject stakes on each gamble test trial for which they had chosen the more likely outcome), (3) deliberation time (average response time to make the probability decision), and (4) risk adjustment (the rate at which participants increase their bets in response to the more favorable ratios blue/red). Healthy controls usually adjust their bet according to the ratio of red and blue boxes; that is, betting fewer points if the odds of winning are lower. Finally, a comparison of the proportion of points bet in the ascending and descending condition enables an assessment of delay aversion. In particular, delay-aversive individuals will place low bets in the ascending condition, coupled with high bets in the descending condition. In contrast, individuals with a preference for risk will typically delay their response to place high bets in the ascending condition.

### Operational definition of treatment drop-out

Duration of treatment in TCs can range from 6 months up until 2 years. Different from our previous study in CDI (19), we coded treatment retention in the present study as a binary variable: treatment completion vs. drop-out. More specifically, we differentiated those participants who completed treatment in the TC and all of the objectives that were laid out at the beginning of treatment (treatment completers) from those that left the program prematurely (drop-outs).

### Statistical analysis

Data were first screened for normality and univariate outliers. Differences between treatment completers and drop-outs on demographic, drug use and decision-making variables were tested using independent sample *t*-tests for continuous variables (e.g., years of education) and chi-square analyses for categorical data (e.g., gender). In order to examine whether treatment completers and drop-outs differed in decision-making performance, we performed Block*Group mixed-design ANOVAs for the IGT (Block-by-Block) and Condition*Group designs for the CGT (Ascending vs. Descending conditions). When the assumption of sphericity was violated, as assessed using the Mauchly sphericity test, the number of degrees of freedom against which the *F*-ratio was tested was reduced by the value of the Greenhouse–Geisser epsilon (35).

The third set of analyses looked at the degree to which variables that significantly differed between treatment completers and drop-outs predicted treatment drop-out. For these analyses, we used a logistic regression analysis with drop-out as the dependent variable and the main demographic, drug use and decision-making variables as the predictors. Variables significant in the initial (univariate) regression analyses were simultaneously entered into the final logistic regression model (enter method), designed to determine whether these predictors were independently associated with treatment drop-out. Multicollinearity diagnostic statistics for the logistic model (tolerance values and VIF) were examined to exclude multicollinearity due to interdependency between the predictor variables. We calculated the classification accuracy of the final model. All analyses were performed using SPSS, version 20.0.

## Results

### Participants

A total of 150 patients were included in the present analyses. Results indicated that more than half of the sample dropped out of treatment prematurely (84/150; 56%), compared to 44% (66/150) who completed treatment. The mean length of stay in TC treatment for the entire sample was 150.15 days (SD = 77.04); there were significant differences between the patients who completed treatment and those who did not. In particular, treatment completers had a mean stay of 207.61 days (SD = 64.54), whereas drop-outs had a mean stay of 105 days (SD = 52) (*t* = 10.78, *p* < 0.01). The demographic and drug-related characteristics/differences between treatment completers and non-completers are presented in Table 1. Groups did not differ in terms of gender (χ^2^ = 0.28, df = 1, *p* = 0.60) or years of education (*t* = −0.33, *p* = 0.74). However, treatment completers and drop-outs significantly differed in terms of their mean age, with drop-outs being significantly younger (34.87 ± 8.09) compared to treatment completers (37.73 ± 8.34) (*t* = 2.12, *p* = 0.04). Most drug-related variables did not differ between treatment completers and drop-outs. The only drug-related variable that differed significantly in both groups was the years of regular cocaine use, with drop-outs having a briefer history of regular cocaine use (years) compared to those who completed treatment (15.90 ± 6.95 compared to 18.65 ± 7.82 respectively; *t* = 2.28, *p* = 0.02).

**Table 1:** **Descriptive information for demographic variables, patterns of cocaine, heroin, and other drug use in treatment completers (*N* = 66) and drop-outs (*N* = 84)**.

		Treatment completers (*N* = 66)	Drop-outs (*N* = 84)
Demographics	Gender (% male/female)	94/6	92/8
	Age	37.73 ± 8.34*	34.87 ± 8.09
	Years of education	10.61 ± 2.47	10.74 ± 2.43
Drug use	Cocaine use
	Age of first use	19.08 ± 4.99	18.96 ± 5.07
	Age of onset problem use	22.29 ± 6.18	20.86 ± 5.57
	Years of regular use	18.65 ± 7.82*	15.90 ± 6.95
	Mean use per week (days)	5.02 ± 1.07	5.06 ± 1.01
	Mean amount per use (g)	0.81 ± 0.66	0.82 ± 0.81
	Peak amount per use (g)	2.40 ± 2.17	2.56 ± 2.65
	Route of administration
	Oral (%)	/	1/84
	Sniffed (%)	20/66	23/84
	Injected (%)	10/66	8/84
	Smoked (%)	36/66	51/84
	Inhaled (%)	/	1/84
	Heroin use (71.3%)	45/66 (68%)	62/84 (74%)
	Age of first use	21.53 ± 5.89	20.60 ± 4.72
	Age of onset problem use	23.04 ± 7.01	21.73 ± 6.25
	Years of regular use	12.42 ± 8.35	10.10 ± 7.08
	Mean use per week (days)	4.87 ± 1.39	4.53 ± 1.39
	Mean amount per use (g)	0.39 ± 0.49	0.28 ± 0.28
	Peak amount per use (g)	0.91 ± 0.85	0.70 ± 0.61
	Other drug use past 30 days
	Cannabis	26/66 (39.39%)	29/84 (34.52%)
	Alcohol	36/66 (54.55%)	44/84 (52.38%)
	Stimulants	3/66 (4.55%)	2/84 (2.38%)
	Hallucinogens	1/66 (1.52%)	0/84
	Benzodiazepines	13/66 (19.70%)	21/84 (25%)

*Results shown are mean ± SD (range) or %*.

*p < 0.05.

### Iowa gambling task

**Figure 1:**
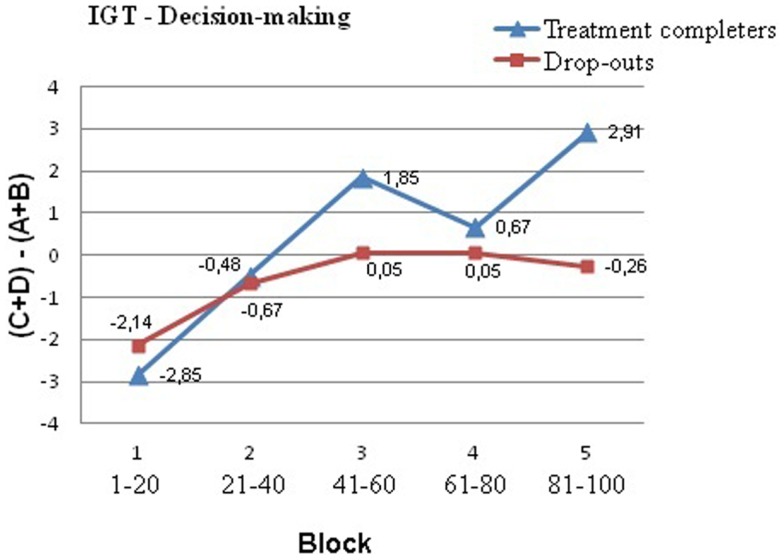
**Performance on the Iowa Gambling Task (IGT) as a function of group (drop-outs vs. treatment completers) and blocks (1–5)**. Each block (1–5) represents 20 sequential card selections. Net score is calculated by subtracting the number of disadvantageous deck selections (A + B) from the number of advantageous card selections (C + D). A negative net score indicates poor decision making. Compared to treatment completers, individuals in the drop-out group tended to select more cards from the risky decks (A and B) than from the safe decks (C and D), although this difference only reached statistical significance in the fifth block (last 20 trials).

Analyzing the IGT-profile of the entire sample using ANOVA repeated measures, we found a significant effect of block [*F*(3.62, 535.08) = 8.46, *p* < 0.01]. The pattern of net score change over block was significantly linear (*p* < 0.01). Overall, these results indicate that participants made more advantageous choices as the task progressed. However, when the effect of block was examined individually for treatment completers and drop-outs (separate repeated-measures ANOVAs for each group), we found that the main effect of block was only significant among treatment completers [repeated-measures ANOVA, effect of block *F*(3.09, 200.77) = 6.90, *p* < 0.01)]. In contrast, the drop-out group did not improve their performance as the task progressed [*F*(4,80) = 1.66, *p* = 0.17] (Figure 1). Results showed a trend for a block*group interaction [*F*(3.62, 535.08) = 2.06, *p* = 0.09]. Pairwise block-by-block between-group comparisons showed that performance of treatment completers and drop-outs significantly differed on the last (fifth) block of the IGT: drop-outs (*M*_net score_ = −0.3) selected significantly more often cards from the disadvantageous decks than treatment completers (*M*_net score_ = 2.9) during this block (*t* = 2.24, *p* = 0.03) (Table 2).

**Table 2:** **Decision-making variables**.

	Treatment Completers (*N* = 66)	Drop-outs (*N* = 84)
IGT
Net scores	2.1 ± 21.8	−3 ± 23.5
Block 1	−2.9 ± 6.3	−2.1 ± 6.3
Block 2	−0.5 ± 6.1	−0.7 ± 6.4
Block 3	1.9 ± 7.2	0.1 ± 7.5
Block 4	0.7 ± 8.6	0.1 ± 7
Block 5	2.9 ± 9.1*	−0.3 ± 8.2
CGT
Quality of decision-making (%)	91.4 ± 9.1*	86.6 ± 13.7
Ascending condition	91.4 ± 9.7	85.3 ± 15.5
Descending condition	91.4 ± 10.8	87.9 ± 15.0
Risk-taking	0.5 ± 0.1	0.5 ± 0.1
Ascending condition	0.4 ± 0.2	0.4 ± 0.2
Descending condition	0.9 ± 0.1	0.9 ± 0.2
Deliberation time (ms)	4506.2 ± 4989.9	4512.5 ± 4352.8
Risk adjustment	1.1 ± 0.8	1.07 ± 0.8

*Results shown are mean ± SD*.

*p < 0.05.

### Cambridge gamble task

#### Quality of decision-making

There was no significant effect of condition [*F*(1,148) = 1.70, *p* = 0.19] on the quality of decision-making. However, we found a statistically significant group effect [*F*(1,148) = 5.89, *p* = 0.02]. Whereas a *post hoc* analysis showed that, compared to treatment completers, the drop-out group made poorer decisions in the ascending condition (*t* = 2.78, *p* < 0.01) (see Table 2), group by condition interaction was not significant [*F*(1,148) = 1.81, *p* = 0.18].

#### Deliberation time

Deliberation time was not affected by condition [*F*(1,148) < 1, *p* = 0.58] and between-subject analysis did not reveal a group effect [*F*(1,148) < 1, *p* = 0.99].

#### Risk-taking

A mixed-model ANOVA of betting data identified a significant main effect of condition [*F*(1,148) = 227.46, *p* < 0.01], as subjects placed larger bets in the descending (mean 67%) than in the ascending condition (mean 41%). There was no significant effect of group (treatment completers and drop-outs did not differ in the mean proportion of total points they staked on each gamble test trial for which they had chosen the more likely outcome) [*F*(1,148) < 1, *p* = 0.77] and group by condition (ascending vs. descending) interaction terms were not significant [*F*(1,148) = 1, *p* = 0.32]. This finding suggests that both groups did not differ in their tendency to take an early bet, which provides an index of impulsivity or delay aversion.

#### Risk adjustment

A mixed-model ANOVA of risk-adjustment data identified a significant main effect of condition [*F*(1,148) = 20.75, *p* < 0.01], with subjects showing more adjustment of their bets in the ascending condition. There was no significant effect of group [*F*(1,148) < 1, *p* = 0.66] or group*condition interaction [*F*(1,148) < 1, *p* = 0.99]. As such, there were no differences between treatment completers and drop-outs in the extent to which they adapted their bets according to the ratio of colored boxes.

### Prediction of treatment drop-out

Variables that significantly differed between treatment completers and drop-outs were tested for their predictive capacity. For the demographical and drug-related variables, these were age and years of regular cocaine use (see Table 1). For the decision-making variables, we included performance on block 5 of the IGT (as block-by-block comparison showed significant differences between treatment completers and drop-outs on this block, see Iowa Gambling Task) and mean scores on CGT quality of decision-making (as a repeated measure ANOVA showed a significant group effect on the quality of decision-making, see Cambridge Gamble Task). Initial analyses of the data seemed to support the idea that age (χ^2^ = 4.48, df = 1, *p* = 0.03), years of regular cocaine use (χ^2^ = 5.16, df = 1, *p* = 0.02), IGT net scores on block 5 (χ^2^ = 4.96, df = 1, *p* = 0.03) and CGT quality of decision-making (χ^2^ = 6.29, df = 1, *p* = 0.01) were all significant predictors of treatment drop-out. Due to the high correlations between age and years of regular cocaine use (*r* = 0.80, *p* = 0.01), age was not retained for multivariate regression. A logistic regression analysis was conducted to predict treatment drop-out using years of regular cocaine use, IGT net scores on block 5, and CGT quality of decision-making as predictors. Collinearity statistics for the predictor variables yielded tolerance values between 0.94 and 0.99 and all VIF values were below 10, indicating that the validity of the regression model was not threatened by multicollinearity. A test of the full model against a constant only model was statistically significant, indicating that the predictors as a set reliably distinguished between treatment completers and drop-outs (χ^2^ = 13.51, df = 3; *p* < 0.01). Nagelkerke’s *R*^2^ of 0.12 indicated that the three predictors explained about 12% of the total variance in treatment drop-out. Prediction success for drop-out was 75%. The Wald criterion demonstrated that only the two decision-making variables made a significant (independent) contribution to prediction (*p* = 0.05) (Table 3). A stepwise backward regression (likelihood ratio test) showed that the goodness of fit of the model did not change significantly when years of regular cocaine use was removed. Removing this variable from the initial model moreover slightly improved the classification accuracy of drop-outs (from 75 to 77.5%). The standardized beta-coefficients, Wald statistics and significance levels for the predictors included in the two models are displayed in Tables 3 and 4.

**Table 3:** **Multivariate prediction of treatment drop-out with a logistic regression model**.

Predictors	*B*	SE	Wald statistics	*p*-Value
Years of regular cocaine use	−0.04	0.02	2.63	0.10
IGT block 5	−0.04	0.02	3.78	0.05
CGT quality of decision-making	−3.28	1.71	3.68	0.05

**Table 4:** **Final prediction model**.

Predictors	*B*	SE	Wald statistics	*p*-Value
IGT block 5	−0.04	0.02	4.35	0.04
CGT quality of decision-making	−0.04	0.02	5.01	0.02

## Discussion

The present study is the first to examine the relationship between two validated tasks of decision-making and treatment drop-out in a relatively large (*n* = 150) and unselected sample of primarily CDI enrolled in long-term residential TCs. Our main finding is that performance on two tasks of decision-making, the IGT and CGT, was significantly related to and predictive of treatment drop-out. Results suggest that after entering long-term residential treatment for cocaine dependence, intact decision-making processes may be crucial to adhere to treatment and complete treatment objectives.

In general, individuals choose increasingly from the advantageous decks as the IGT progresses (20, 24). In corroboration with this normative trend, our sample showed improvements over the course of the tasks as an entire group. However, when split into treatment completers and drop-outs, we found that only treatment completers showed an improvement as the IGT progressed (these individuals ultimately had positive “money” gains). In contrast, the drop-out group did not select more frequently from the advantageous decks, ultimately lost “money” and displayed minimal evidence of learning to select from the advantageous decks across the task, as suggested by their (still) negative net scores on block 5 of the IGT (last 20 trials). Conceptually, the later blocks of the IGT have been suggested to represent post-learning stages (players presumably have developed explicit knowledge of the risk profile across IGT alternatives) and performance on these blocks is believed to reflect decision-making under risk (rather than ambiguity) (36). This notion has been supported by a number of studies pointing to significant correlations between later stage IGT selections and an individual’s propensity for deliberate risk-taking (36, 37). However, recent evidence indicates that these correlations may not be present among high-impulsive individuals, potentially suggesting that this group fails to develop explicit knowledge of risky IGT alternatives (37).

The finding that drop-outs failed to develop a preference for the advantageous decks and continued to select cards from the bad decks, despite being penalized, may suggest several things. First, this group may be less sensitive to or may fail to generate emotion-related signals (somatic markers) to losing (38). These somatic markers normally facilitate advantageous decisions by steering away from options that, through prior experience, are associated with unpleasant gut feelings (39). Hypothetically, weaker somatic signals to negative outcomes in CDI may lead them to be less hesitant about terminating treatment prematurely. However, a number of alternative theories have been proposed to explain impaired decision-making in drug-dependent individuals, including poor working memory and cognitive flexibility (40). As the fixed card order on the IGT induces an initial preference for the ultimately risky decks, disadvantageous performance in the drop-out group may reflect a difficulty in reversing behaviors that may once have been rewarding but ultimately bring high costs, such as continued drug use. Corroborating this notion, significant associations between poor decision-making on the IGT and difficulties with achieving and maintaining abstinence have been reported among individuals dependent on cocaine, opiates, and alcohol (30–32, 41, 42).

Using an alternative task of decision-making (CGT) that is not confounded by information processing demands, we were able to show that drop-outs were less likely to choose the most favorable option (i.e., the box color in the majority) compared to treatment completers. These choices reflect low quality decisions, given that the probabilities associated with each choice are visible at the time of the decision. As both groups equally used the box ratio information about outcome probability to adjust their bets (as shown by the absence of significant differences in adjustment slopes across groups), findings indirectly suggest that the lower-quality decisions in the drop-out group cannot be attributed to poor processing of probability information. A comparison of betting behavior in the ascending and descending condition between drop-outs and treatment completers further suggests that poor decision-making in the drop-out group was not due to greater delay aversion or impulsivity. In fact, both groups showed evidence of impulsive responding, as indicated by the significantly higher bets placed in the descending condition. Finally, the absence of differences between both groups in terms of deliberation time argues against an explanation in terms of speed-accuracy. Overall, our findings suggest that drop-out vulnerable cocaine users fail to integrate prior experiences into their decisions or neglect probability information, thus ignoring the broader context in which decisions are made. These deficits may be associated with alterations in the orbitofrontal and the ventromedial prefrontal cortex (regions implicated in the use of feedback to improve decision-making) or the dorsolateral prefrontal loop, which has a critical role in overseeing subordinate processes through the exercise of executive control (36, 43).

### Clinical implications

Our findings have important clinical implications. If replicated, the present results suggest that (1) tasks indexing decision-making may be added to the range of clinical information that is collected at treatment intake in order to identify CDI who are at risk for premature treatment drop-out and that (2) treatment drop-out among CDI admitted to TCs may be reduced by targeting cognitive and affective processes involved in decision-making.

In line with the multiple processes implicated in the regulation of decision-making, the integrity of prefrontal cortical, and executive functioning in general and aspects involved in risk-reward decision-making (executive functioning, reversal learning and interoceptive awareness) in particular represent interesting targets for consideration (44). Whereas more research is needed in order to examine the feasibility of incorporating these interventions into real-world clinical settings, preliminary evidence suggests that a combination of executive functioning training (e.g., Goal Management Training) and mindfulness-based meditation (45) and/or emotion regulation techniques (46, 47) may have the potential to improve adaptive decision-making in drug users. Importantly, these strategies should be modified and employed in a manner that specifically appeals to or targets cognitively impaired subgroups of drug users. Indeed, some of these interventions assume a certain level of cognitive ability needed to acquire skills, such that patients who are substantially cognitively impaired may be less likely to benefit from them. Similarly, neurocognitive dysfunctions, including disadvantageous decision-making, have been linked to both structural and functional brain alterations, which are likely to compromise learning and successful behavioral modification during treatment (48). Therefore, pharmacological interventions or neuromodulation-based approaches (e.g., Transcranial Magnetic Stimulation) aimed at upregulating brain functioning (48–50) may provide neurocognitively impaired drug users with a stronger ability to benefit from cognitively oriented treatment programs. Modafinil for example, could act as a successful adjunct for increasing the effectiveness of executive training programs in cognitively impaired drug users by boosting neural functioning in regions implicated in learning and cognitive control (i.e., insula, ventromedial prefrontal, and anterior cingulate cortices). However, the effectiveness of combining these approaches has yet to be systematically explored and reported on and might be a promising area for future research.

### Study limitations

Although we believe that the current study has important clinical implications, several limitations should also be noted. First, several factors should be considered before generalizing from our findings. Specifically, our findings are based on a predominantly male sample of poly-drug-using CDI, the majority of whom were crack users, enrolled in long-term, residential TCs. Drug users admitted to TCs often have relatively severe problems, prior drug abuse treatment experience, and a criminal justice status. As such, the present findings may not extrapolate to other treatment samples (e.g., women, individuals enrolled in outpatient treatment settings). Still, it should be noted that our sample represents a group of CDI encountered in real clinical contexts, which increases the ecological validity of the study results. Despite our finding that two indices of decision-making predicted treatment drop-out, there was a significant amount of variance that was not accounted for by the variables examined in this study. Importantly, we did not take into account the effects of other potentially relevant person-related factors, such as psychiatric comorbidity, personality (e.g., impulsivity, perseverance), or intellectual functioning (51–54). Further, drop-out from treatment is not driven purely by person-related factors (actually, person-related variables typically predict only a small proportion of the variance in drop-out), but also varies as a function of treatment-related variables and interactions between the individual and the treatment environment (30, 31, 55).

We did not examine potential mediators of both cognitive performance and treatment retention. Among many other factors, motivation may have functioned as a mediator of both apparent cognitive performance as well as treatment retention: motivation has been shown to be an important factor in treatment retention among substance-dependent individuals (56–58) and lower motivation to change has been found to correlate with poorer performance on a task of decision-making (59). As such, it is possible that the observed differences in cognitive task performance between treatment completers and drop-outs reflect a difference in motivation for treatment and in the motivation to perform well on the decision-making tasks. Also, our data do not exclude the possibility that motivation for treatment or motivation to change functioned as a mediator of the relationship between disadvantageous decision-making and treatment drop-out. Indeed, the way in which neurocognitive dysfunctions impact upon treatment outcomes may not necessarily be direct (60). Rather, neurocognitive impairments can impede treatment outcomes through their effects on treatment processes or more intrapersonal factors (60). For example, poor neurocognitive functioning has shown significant associations with lower motivation to change or poorer self-efficacy in treatment samples of alcoholics (61, 62). These countervailing effects of neurocognitive dysfunctions on intrapersonal processes may cancel out when analyzing direct effects of impairment on treatment drop-out. Future studies may help to better understand the nature of the current findings by examining a range of potential mediators, including motivation.

In summary, the present study is the first to show that primarily CDI who drop-out of residential treatment prematurely fail/neglect to integrate prior experiences/knowledge regarding outcome probabilities into their decisions. Further, our findings indirectly suggest that previous studies may have failed to find associations between IGT performance and treatment retention because early and late IGT selections were combined into a single measure and changes in task performance were not taken into account. Whereas the precise underlying processes contributing to disadvantageous decision-making patterns remain to be explored, our findings have potential implications for the treatment of cocaine dependence.

## Conflict of Interest Statement

The authors declare that the research was conducted in the absence of any commercial or financial relationships that could be construed as a potential conflict of interest.
